# Pre‐illness dietary risk factors in dogs with chronic enteropathy

**DOI:** 10.1111/jvim.16872

**Published:** 2023-09-24

**Authors:** Isla Trewin, Aarti Kathrani

**Affiliations:** ^1^ Royal Veterinary College University of London London England

**Keywords:** canine, carbohydrate, gastrointestinal, ingredients, moisture, protein

## Abstract

**Background:**

Dietary factors have been extensively studied as potential triggers of inflammatory bowel disease in humans. Scant literature exists regarding diet as a pre‐illness risk factor in dogs with chronic enteropathy (CE).

**Hypothesis:**

To evaluate possible pre‐illness dietary risk factors in dogs with CE.

**Animals:**

Ninety‐five client‐owned dogs; 48 with CE (25 presumptive and 23 confirmed) and 47 without a history of signs of gastrointestinal disease.

**Methods:**

Retrospective case‐control questionnaire‐based study at a veterinary referral teaching hospital in the United Kingdom. Diet history was obtained relating to the onset of initial presenting signs for all dogs. The main diet consumed underwent ingredient analysis and caloric distribution calculation using a guaranteed analysis convertor software. Length of time the main diet was fed and adherence to the World Small Animal Veterinary Association Global Nutrition Committee guidelines was also recorded.

**Results:**

The frequency of the main diet containing no carbohydrate was greater for controls (5/47 dogs, 11%) vs the combined presumptive and confirmed CE dogs (0/48 dogs, 0%; *P* = .05). Fewer dogs with confirmed CE were fed a main diet containing red meat as the primary protein source (2/23 dogs, 9%) vs controls (15/47 dogs, 32%; *P* = .03). A main diet moisture percentage of ≤14% as fed was significantly associated with confirmed CE in logistic regression analysis (OR 5.71 [95% CI: 1.18‐27.69]; *P* = .03).

**Conclusions and Clinical Importance:**

The presence of dietary carbohydrate, protein source, and dietary moisture content, or factors related to moisture content such as preservatives, might play a role as potential pre‐illness dietary risk factors in dogs with CE.

**Abbreviations**
AGEs: advanced glycation end products
BCS: body condition score
CD: caloric distribution
CE: chronic enteropathy
CHO: carbohydrate
CI: confidence interval
CMC: carboxymethylcellulose
DHA: docosahexaenoic acid
EPA: eicosapentaenoic acid
FRE: food‐responsive enteropathy
IBD: inflammatory bowel disease
P80: polysorbate‐80
SCFA: short‐chain fatty acids
WSAVA GNC: World Small Animal Veterinary Association Global Nutrition Committee

## 1 INTRODUCTION

The cause of chronic enteropathy (CE) in dogs is multifactorial, with a growing body of evidence supporting the role of the immune system, genetics, and microbiota in its etiopathogenesis.^1^, ^2^, ^3^ Literature examining the contribution of environmental factors in the development of CE in dogs is sparse. Cats fed a commercial diet that did not meet the World Small Animal Veterinary Association (WSAVA) Global Nutrition Committee (GNC) recommendations during early life are more likely to develop signs of gastrointestinal disease.^4^ A diet low in carbohydrate and high in fat is associated with a lower risk of developing chronic signs of gastrointestinal disease in adult dogs, but the role of dietary ingredients, or the diet at the onset of clinical signs is unclear.^5^

Inflammatory bowel disease (IBD) in humans is a chronic progressive condition, comprised of Crohn's disease and ulcerative colitis.^6^ The complex etiology associated with these conditions involves the interplay between environmental factors, gastrointestinal microbiome, genetics, and the immune response.^7^ There is an increasing incidence of IBD in association with lifestyle changes in Westernized countries, specifically dietary patterns.^8^ As a result, diet has been intensively studied to examine its role in the development of IBD. These studies demonstrate that a greater intake of animal protein, particularly red meat, is significantly associated with the development of IBD and relapses in ulcerative colitis.^9^, ^10^, ^11^ Large quantities of specific dietary fats and the presence of food additive emulsifiers in murine and in vitro studies are implicated in the pathogenesis of ulcerative colitis and IBD, respectively.^12^, ^13^, ^14^ A high intake of dietary fiber is protective against Crohn's disease.^15^

To the authors' knowledge, no previous studies have evaluated ingredients or macronutrient profiles at the onset of initial clinical signs to further assess potential pre‐illness dietary risk factors in dogs with CE. The aim of this study was to assess the caloric distribution (as percentage fat, protein, and carbohydrate on a metabolizable energy basis), alongside other dietary factors such as ingredients of the main diet being fed at the onset of signs of gastrointestinal disease and their association with CE. The identification of specific dietary risk factors would be beneficial in the prevention of disease and provide further understanding regarding the etiopathogenesis of CE in dogs. We hypothesized that the dietary composition of the main diet fed at the onset of initial presenting signs is significantly different between dogs with CE and those without a history of gastrointestinal disease.

## 2 MATERIALS AND METHODS

### 2.1 Case selection and data collection

This was a retrospective case‐control questionnaire‐based study performed at a referral teaching hospital in the United Kingdom between August 2020 and January 2022. One hundred dogs were recruited: 50 in the CE group and 50 in the control group. The CE group included 25 dogs with a confirmed diagnosis of CE and 25 dogs with a presumptive diagnosis of CE. Sample size was based on the number of dogs typically examined with suspected or confirmed chronic enteropathy at the referral teaching hospital during the timeline for recruitment of 18 months. Dogs were included if they had a duration of gastrointestinal disease signs ≥3 weeks or biochemical and histopathological findings consistent with inflammatory protein‐losing enteropathy regardless of duration. Dogs in the presumptive group required the following diagnostic investigations: (1) complete blood count, (2) serum biochemistry, (3) basal cortisol in the absence of a neutrophilia or lymphopenia, (4) serum vitamin B12 concentration, (5) fecal parasitology or fenbendazole course, and (6) abdominal ultrasound or CT. Those dogs classified as having a confirmed diagnosis of CE additionally underwent gastrointestinal biopsies and had consistent histopathologic findings. All dogs with protein‐losing enteropathy had urinalysis performed. The control group included dogs that presented to the same Internal Medicine Service with no current or historical signs of gastrointestinal disease for routine consultation during the same time period. These dogs were randomly selected for inclusion.

Online questionnaires were sent to participating owners, requiring a full diet history relating to the point of onset of initial clinical signs for both groups (Data S1). The information requested consisted of name of diet, brand, formulation, flavor, length of time the specific diet had been fed before the onset of initial clinical signs, the amount of diet being fed per day and treat provisions. Where multiple diets were fed, owners were asked to identify the main diet by confirming which diet made up the largest proportion of the daily caloric intake. A follow‐up telephone call was carried out if the questionnaire was incorrectly completed or where there was a lack of response. Where owners could not accurately recall previous feeding patterns, subjects were excluded because of incomplete diet history. For those dogs meeting the inclusion criteria, records were reviewed for signalment, type and duration of clinical signs, and results of diagnostic investigations.

### 2.2 Dietary analysis

The main diet consumed at the onset of initial presenting clinical signs underwent the following analysis.

#### 2.2.1 Dietary formulation and processing

Categorization of the main diet was carried out based on formulation (traditionally processed, home cooked, and raw) and dietary processing (extrusion, canning, and minimal processing, the latter including all raw and home cooked diets).

#### 2.2.2 Ingredient analysis

The main carbohydrate source, defined as the first listed on the ingredient panel, was recorded and categorized into 1 of the following groups: cereals/grains, vegetables/legumes, and no carbohydrate source. All vegetables were included in the vegetables/legumes category, including processed vegetable products, and none of these diets had cereals/grains listed lower down their ingredients list. The main diet was also classified according to whether it contained wheat or not.

The main protein source was also defined as the first listed on the ingredient panel and underwent categorization into either animal or plant origin. The first protein source was characterized as poultry, red meat, fish, vegetarian, or mixed meats, where multiple flavors of the same diet were fed. For those diets containing red meat as the first protein source, this was further characterized as beef, lamb, pork, or venison. Each main diet was also assessed for a source of dairy, which included the presence of milk or cheese.

The presence of certain preservatives and emulsifiers known to incite gastrointestinal inflammation in either animal or in vitro studies (carrageenan, carboxymethylcellulose [CMC], and polysorbate‐80 [P80]^16^, ^17^) was also documented.

Sources of the omega‐3 fatty acids eicosapentaenoic acid (EPA) and docosahexaenoic acid (DHA) were also recorded, as determined by the inclusion of whole fish, fish oil, EPA, DHA, and exogenous omega 3 in the listed ingredients for the main diet. Fish meal was not included in the search terms, as this would typically be defatted and therefore not contribute EPA or DHA, however, this was present in 1 diet, although alongside whole fish as the main protein source. Algae and algae oil were included in the search terms (considered an alternative source of omega‐3 fatty acids EPA and DHA), however were not present in any of the diets.

The presence of pre/probiotics, using the search terms fructooligosaccharides (FOS), mannan oligosaccharides (MOS), and *Enterococcus faecium* in the ingredient panel was also recorded.

#### 2.2.3 Caloric distribution

Caloric distribution of the main diet was calculated using a guaranteed analysis convertor software (www.BalanceIT.com, accessed dates: August 2020 to January 2022). As such, supplemental diets and treat provisions listed by owners were not included in the analysis. The caloric distribution, defined as the percentages of fat, protein, and carbohydrate on a metabolizable energy basis, was calculated using Modified Atwater factors utilized as part of the software. The crude protein, crude fat, crude fiber, moisture, and crude ash percentages as fed, were recorded from the analytical constituents provided for the main diet. Manufacturers were contacted and the moisture content requested on an as fed basis when this was absent (eg, <14%). Crude ash was available for all analyses, and therefore a default estimation was not utilized.

#### 2.2.4 Compliance with WSAVA GNC guidelines

Finally, all pet food manufacturers were contacted and asked a series of questions to confirm whether diets complied with the WSAVA GNC guidelines (Data S2).^18^

#### 2.2.5 Exclusion criteria

Dogs that were fed multiple diets where the owner could not identify a main formulation were excluded from analysis (n = 5). In the instance of different flavors of the same diet being fed, the difference in caloric distribution and crude fiber, moisture, and crude ash percentage as fed was assessed between formulations. If the difference exceeded 5% for any of the aforementioned variables, dogs were excluded from the statistical analysis and only ingredient analysis of the main diet was assessed (n = 3). Where this difference was <5%, an average was utilized for the final statistical analysis (n = 3). Home‐cooked or home‐prepared diets were also excluded from the statistical analysis, as the level of detail provided on the questionnaire did not allow for an accurate calculation of the caloric distribution; however, ingredient analysis was carried out (n = 3). Finally, diets where proximate analysis were unavailable from the manufacturer were also excluded from statistical analysis and only underwent ingredient analysis (n = 1). Further information regarding the exclusion of these dogs is provided in Data S3.

### 2.3 Statistical analysis

A commercially available computer software package (IBM SPSS Statistics Version 28) was utilized for all statistical analyses. Histograms and the Shapiro‐Wilk test were performed to assess for normality of continuous variables. Results were reported as mean (SD) if normally distributed and median (range) if not normally distributed.

Two stages of statistical analysis were performed; the first compared the controls and combined (presumptive and confirmed) CE cases (primary analysis) and the second compared the controls and confirmed CE cases only (secondary analysis). In the primary analysis, Chi‐squared was utilized to analyze categorical data including body condition score, sex, neuter status, diet processing, main/first meat source, wheat source, WSAVA GNC adherence, omega 3 presence, and pre/probiotic presence. Diet formulation, protein source, and carbohydrate source were analyzed using the Fisher exact test. In the secondary analysis, the same statistical tests were performed for the aforementioned variables, except for diet processing and wheat source, which were analyzed using a Fisher exact test. For both the primary and secondary analysis, continuous data including age, body weight, length of time main diet was fed before the onset of initial clinical signs, percentage fat/protein/carbohydrate on a metabolizable energy basis, percentage crude fiber, moisture, and crude ash as fed were analyzed using the Mann‐Whitney *U* test.

Caloric distribution, percentage crude fiber, moisture, and crude ash as fed were assessed as both continuous (Mann‐Whitney *U* test) and categorical variables (Chi‐squared). Caloric distribution represents the percentage metabolizable energy contributed by the 3 macronutrients (protein, fat, and carbohydrate) within a diet. Categorization was either based on biologically relevant grouping or binary separation via the median.

Binary logistic regression was carried out to assess the relationship of age, length of time the diet was fed before onset of initial clinical signs, carbohydrate source, caloric distribution, percentage crude fiber as fed, percentage moisture as fed, and percentage crude ash as fed between CE and control groups. Age and length of time diet was fed were analyzed both as continuous and categorical variables, categorization was again based on biologically relevant grouping. Multivariable analysis was pursued for variables which were significant in the logistic regression and considered to be confounding (primary analysis; age and length of time diet was fed). As part of this multivariable analysis, age and length of time diet was fed were both assessed as binary variables, with categorization based on the median age and the delineation of 6 months, respectively.

A type I error rate of 0.05 was utilized for all statistical analyses.

## 3 RESULTS

A total of 100 dogs were enrolled to the study; demographics of these dogs are presented in Data S4.

At the onset of initial clinical signs, the majority of dogs were fed a traditionally processed cooked diet (41/47 control dogs, 44/48 CE dogs). Two CE dogs and no control dogs received a home‐cooked diet. Two CE dogs and 6 control dogs received a raw food diet, 6/8 of these raw food diets had undergone freezing and 2/8 had not had any traditional processing. Meat was the primary protein source in most diets (92 dogs), with only 3 vegetable protein source diets (2 control dogs, 1 CE dog). These 3 diets did however contain meat/animal derivatives lower down the ingredient list and therefore were not vegetarian or vegan. Further information regarding the protein‐source of these diets is available in Data S5. None of the main diets contained a dairy source or preservatives such as carrageenan, CMC, and P80.

For the 92 dogs receiving a commercial main diet, 75/92 (82%) utilized a typical analysis. Seventy‐one of these diets listed analytical constituents however, this was assumed to represent typical analysis because of European Pet Food Industry Federation (FEDIAF) legal requirements. Guaranteed analysis was reported by the manufacturer in 17/92 (18%) dogs.

The combined presumptive and confirmed CE dogs were significantly younger (median 3 year 6 months, range 3 months to 10 years) compared with the control group (median 7 years 7 months, range 7 months to 15 years 7 months; *P* < .001). This was similarly the case for confirmed CE dogs (median 5 years, range 6 months to 10 years 1 month; *P* = .01). For both the primary (controls vs combined presumptive and confirmed CE group) and secondary (controls vs confirmed CE group) analysis, there was no significant difference in body weight between groups (primary analysis *P* = .85, secondary analysis *P* = .87). The body condition score of CE dogs was significantly lower in both the primary (*P* ≤ .001) and secondary (*P* ≤ .001) analysis. Sex and neuter status did not differ significantly between groups in either the primary (sex; *P* = .62, neuter status; *P* = .94) or secondary analysis (sex; *P* = .53, neuter status; *P* = .76). The main diet was fed for a significantly shorter duration of time before onset of initial clinical signs in the CE dogs (median 12 months, range 0.5‐84), compared with controls (median 36 months, range 1‐96; *P* < .001). However, when length of time diet was fed was standardized to age (length of time diet fed/age), it was not significant (*P* = .26). In the univariable analysis, dogs fed a consistent diet for >6 months had a significantly lower risk of developing CE (OR = 0.23 [95% CI: 0.09‐0.62); *P* = .004). Older dogs (over the age of 5 years 3 months [median]) also had a significantly lower risk of developing CE (OR = 0.21 [95% CI: 0.09‐0.51]; *P* = .01). In the multivariable analysis, only age remained an independent risk factor after adjusting for duration of diet (OR = 0.22 [95% CI: 0.09‐0.52]; *P* = <.001). Data for statistically nonsignificant variables is provided in Data S6 and Table 1.

**TABLE 1:** Results from statistical analysis of pre‐illness dietary risk factors in dogs with chronic enteropathy (CE).

		Primary analysis^a^			Secondary analysis^b^		
Factor	Category	Control (n = 47)	Cases (n = 48)	*P*‐value	Control (n = 47)	Cases (n = 23)	*P*‐value
Dietary formulation	Traditionally processed	41 (87%)	44 (92%)	.13	41 (87%)	22 (96%)	.06
	Home cooked	0 (0%)	2 (4%)		0 (0%)	1 (4%)	
	Raw	6 (13%)	2 (4%)		6 (13%)	0 (0%)	
Diet processing	Extrusion	30 (64%)	32 (67%)	.78	30 (64%)	20 (87%)	.17
	Canning	11 (23%)	12 (25%)		11 (23%)	2 (9%)	
	Minimal processing	6 (13%)	4 (8%)		6 (13%)	1 (4%)	
Protein source	Animal based	45 (96%)	47 (98%)	.62	45 (96%)	22 (96%)	1.00
	Plant based	2 (4%)	1 (2%)		2 (4%)	1 (4%)	
Main/first meat source	Non‐red meat	32 (68%)	40 (83%)	.08	32 (68%)	21 (91%)	.03
	Red meat	15 (32%)	8 (17%)		15 (32%)	2 (9%)	
Carbohydrate source	Cereals and grains	28 (60%)	28 (58%)	.05	28 (60%)	16 (70%)	.36
	Vegetables and legumes	14 (30%)	20 (42%)		14 (30%)	7 (30%)	
	No carbohydrate source	5 (11%)	0 (0%)		5 (11%)	0 (0%)	
Wheat source	Yes	9 (19%)	7 (15%)	.55	9 (19%)	2 (9%)	.32
	No	38 (81%)	41 (85%)		38 (81%)	21 (91%)	
Adherence to WSAVA GNC guidelines	Yes	18 (38%)	24 (50%)	.25	18 (38%)	14 (6%)	.08
	No	29 (62%)	24 (50%)		29 (62%)	9 (39%)	
Omega 3 source	Yes	30 (64%)	38 (79%)	.10	30 (64%)	19 (83%)	.11
	No	17 (36%)	10 (21%)		17 (36%)	4 (17%)	
Exogenous pre/probiotics	Yes	16 (34%)	16 (33%)	.94	16 (34%)	6 (26%)	.50
	No	31 (66%)	32 (67%)		31 (66%)	17 (74%)	

Abbreviation: WSAVA GNC, World Small Animal Veterinary Association Global Nutrition Committee.

^a^ Evaluation of presumptive and confirmed CE cases vs controls.

^b^ Evaluation of confirmed CE cases vs controls.

For the primary analysis, only the frequency of the main diet containing no carbohydrate was significantly greater for controls (5/47 dogs) compared with the combined presumptive and confirmed CE dogs (0/48 dogs; *P* = .05; Table 1). Post hoc pairwise comparisons were carried out comparing individual carbohydrate sources; vegetables/legumes vs no carbohydrate source (*P* = .01), cereals/grains vs no carbohydrate source (*P* = .03) and cereals/grains vs vegetables/legumes (*P* = .42). Further dietary analysis of the 5 dogs receiving a main diet without a carbohydrate source confirmed that carbohydrate was present in the additional diets and treat provisions being fed. Two of the diets in the vegetable and legumes group contained processed vegetable products, defined as “vegetable derivatives.”

For the secondary analysis, only the presence of a primary red meat source in the diet was significantly different, with fewer confirmed CE dogs being fed a main diet containing red meat as the first protein source (2/23 dogs, 9%) compared with control dogs (15/37 dogs, 32%; *P* = .03; Table 1). There was no difference in the frequency of the main diet containing no carbohydrate between the control dogs and confirmed CE cases in the secondary analysis (*P* = .36). Univariable logistic regression analysis (Table 2) demonstrated that a moisture percentage ≤14% as fed, was significantly associated with confirmed CE (OR 5.71 [95% CI: 1.18‐27.69]; *P* = .03).

**TABLE 2:** Univariable logistic regression results for pre‐illness dietary risk factors in dogs with chronic enteropathy (CE).

		Primary analysis^a^				Secondary analysis^b^				
Factor	Category	Control^c^	Cases^c^	Odds ratio (95% CI)	*P*‐value	Category	Control^c^	Cases^c^	Odds ratio (95% CI)	*P*‐value
CD fat percentage^d^	≤36%	25 (58%)	19 (42%)	Reference	.14	≤33%	22 (51%)	12 (55%)	Reference	.80
	>36.1%	18 (42%)	26 (58%)	1.91 (0.82‐4.43)		>33.1%	21 (49%)	10 (45%)	0.87 (0.31‐2.45)	
CD protein percentage^d^	≤26%	23 (53%)	21 (47%)	Reference	.52	≤25.9%	22 (51%)	11 (50%)	Reference	.93
	>26.1%	20 (47%)	24 (53%)	1.31 (0.57‐3.04)		>26%	21 (49%)	11 (50%)	1.05 (0.38‐2.93)	
CD carbohydrate percentage^d^	≤37%	17 (40%)	27 (60%)	Reference	.06	≤40%	22 (51%)	12 (55%)	Reference	.80
	>37.1%	26 (60%)	18 (40%)	0.44 (0.19‐1.02)		>40.1%	21 (49%)	10 (45%)	0.87 (0.31‐2.45)	
Crude fiber percentage as fed^d^	≤2.5%	25 (56%)	27 (59%)	Reference	.76	≤2.5%	25 (56%)	9 (41%)	Reference	.26
	>2.6%	20 (44%)	19 (41%)	0.88 (0.38‐2.02)		>2.6%	20 (44%)	13 (59%)	1.81 (0.64‐5.08)	
Moisture percentage as fed^e^	≤14%	28 (64%)	32 (70%)	1.3 (0.54‐3.14)	.55	≤14%	28 (64%)	20 (91%)	5.71 (1.18–27.69)	.03
	>14.1%	16 (36%)	14 (30%)	Reference		>14.1%	16 (36%)	2 (9%)	Reference	
Crude ash percentage as fed^d^	≤6.5%	22 (49%)	24 (52%)	Reference	.75	≤7%	29 (64%)	10 (45%)	Reference	.14
	>6.6%	23 (51%)	22 (48%)	0.88 (0.39‐2.00)		>7.1%	16 (36%)	12 (55%)	2.18 (0.77‐6.14)	

Abbreviations: CI, confidence interval; CD, caloric distribution.

^a^ Evaluation of presumptive and confirmed CE cases vs controls.

^b^ Evaluation of confirmed CE cases vs controls.

^c^ Percentage based on the total number of cases where the relevant nutritional information was available.

^d^ Binary categorization based on median value of the variable.

^e^ Binary categorization based on typical upper threshold of dry food moisture percentage as fed (14%).

## 4 DISCUSSION

The existing veterinary literature focuses on the role of diet in the therapeutic management of dogs with CE rather than as a risk factor.^19^, ^20^, ^21^ In addition, most of the literature has predominantly evaluated the role of the immune response, gut microbiota, and genetic predilections in the pathogenesis of this disease.^1^, ^2^, ^3^ To fill this important scientific gap, our study retrospectively evaluated diet as a pre‐illness risk factor for development of CE in dogs. Our study demonstrated that when evaluating all cases of CE, a main diet containing carbohydrate was a potential risk factor in disease development, however, was no longer a risk factor when evaluating confirmed cases of CE only. The primary protein source and a moisture content of ≤14% as fed at the onset of initial clinical signs, were associated with disease development in confirmed cases of CE only. These findings support the idea that diet might play a role in the development of this multifactorial disease process in dogs. In people, the role of diet as a risk factor for IBD has been established and now forms a platform for dietary modification in the prevention and relapse of this disease.^10^, ^22^

In our study, dogs in the combined presumptive and confirmed CE group were younger compared with the control dogs. The dogs in the presumptive CE group were considered most likely to have a food‐responsive enteropathy (FRE) and were placed onto a therapeutic diet trial at the time of enrolment. Food responsive enteropathy is most frequently reported in young dogs and although follow up would be required to confirm a diagnosis of FRE in this subset of dogs, this might account for the significant age difference noted between groups.^20^ Further analysis of defined groups such as FRE and protein‐losing enteropathy (33% of CE dogs) was not pursued as part of this study given the small group sizes and subsequent reduction of statistical power.

We also found that the main diet in the CE group was fed for a significantly shorter duration of time before the onset of signs, however, following multivariable analysis this was no longer significant. Dietary change has an associated effect on the gastrointestinal microbiota, the composition of which plays a key role in the pathogenesis of CE.^23^, ^24^

In our primary analysis, the frequency of the main diet containing no carbohydrate was greater in controls compared with the combined presumptive and confirmed CE dogs. Interestingly, this parallels findings from a study that showed a diet low in carbohydrate during early life is associated with a reduced risk of adult dogs developing chronic signs of gastrointestinal disease.^5^ In human medicine, carbohydrates are implicated in the pathogenesis of IBD, however overall their role is inconsistent in the literature.^25^ Research has focused on available dietary carbohydrate (eg, starch and simple sugars) because of their malabsorption and resulting deleterious effect on the microbiota, alongside the role of resistant starch.^26^ The specific composition of dietary carbohydrate (available vs nonavailable) was not evaluated as part of our study, or the presence of resistant starch. The small cohort size of the subgroup receiving a diet without carbohydrate might have resulted in a type I statistical error, contributing to the significance found in our primary analysis. Unfortunately, the measurement of carbohydrate in pet food inherently presents a challenge given its crude approximation based on the other macronutrient components of the diet.^27^ This generally results in overestimation and could also have affected our findings.

Our study found no association with the presence of carbohydrate in the main diet in dogs with confirmed CE. A few factors could have been responsible for this. First, the suspected CE dogs might have had a nonimmune based intolerance to carbohydrates and therefore were more likely to respond adversely to a diet containing this macronutrient compared with those dogs with confirmed CE. Second, the suspected CE dogs were more likely to have FRE compared with the confirmed CE dogs and therefore, carbohydrates might form more of a role in the pathogenesis of this subgroup of CE. Finally, the confirmed CE group was smaller than the combined suspected and confirmed CE group, and therefore the lack of significance could have been because of a smaller sample size resulting in a type II error.

Our secondary analysis demonstrated that a significantly smaller proportion of dogs with confirmed CE were fed a main diet with red meat as the primary protein source at the onset of initial clinical signs compared with control dogs. In humans, the role of dietary protein source and its potential contribution to the development of IBD is well documented. The findings of our study conflicts the current human literature, which highlights red meat as a risk factor in the development of IBD.^11^ Both red and white meat are implicated as triggers for adverse food reactions in dogs.^28^ Adverse food reactions occur as a result of antigenic stimulation and loss of oral tolerance because of dysregulation of the immune response, a feature which underpins the etiopathogenesis of IBD and CE.^1^, ^28^ Whilst the source of the macronutrient clearly plays an important role, food processing can also greatly alter the configuration of allergens and needs to be taken into account.^29^ Studies also show that additional proteins can be present in foods that were not declared on the ingredient label.^30^, ^31^ Therefore, future studies should look to assess protein sources directly from the food, rather than relying on the ingredient label. This will help to determine the exact role of protein source as a risk factor for CE in dogs.

In our study, main diets fed at the onset of initial clinical signs in dogs with confirmed CE were more likely to have a lower moisture content (≤14% as fed) compared with control dogs. A higher consumption of water is protective in the development of Crohn's disease in children.^32^ The composition of fecal microbiota in people is associated with water intake and as such this might in part explain its protective role in this disease process.^33^ Dry pet food generally has a moisture content of <14% and typically a higher carbohydrate content, both of which were associated with CE in our study. Similarly, given that lower moisture diets are typically those manufactured via the extrusion process, the correlation found in this study might also reflect a consequence of this processing method, such as increased amounts of advanced glycation end products (AGEs). These AGEs have a deleterious effect on the gut microbiota in vivo and are also implicated in the development of inflammatory conditions in people.^34^ Additional contributors, alongside moisture and carbohydrate content, might be the presence of preservatives in dry food. Preservatives have been associated with alterations in gut microbiota in various in vivo studies.^35^ These additives are found in higher quantities in dry pet food preparations, compared with wet, to prevent oxidation and food spoilage. Preservatives were not specifically examined as part of our study because their role in the development of human IBD is not well defined, unlike specific emulsifiers. Emulsifiers associated with deleterious effects on the gastrointestinal tract were not identified in any of the diets in our study.

Dietary fiber is protective against the development of IBD in people.^15^, ^36^ This effect likely stems from colonic fermentation of soluble fiber, resulting in the production of short‐chain fatty acids (SCFA).^37^ These SCFA exert beneficial immunomodulatory effects on the gut.^38^ In our study, crude fiber as fed did not demonstrate a protective effect on the development of CE. Crude fiber, however, only measures a proportion of dietary fiber and does not reflect the total fiber content of a diet.^39^ Nor does it measure any soluble fiber and therefore does not reflect the fermentable fiber content within a diet. This might have led to underestimation of dietary fiber, resulting in a lack of significance.

In our study, each diet was individually analyzed, and the caloric distribution calculated on the basis of the manufacturer's proximate analysis. The type of analysis provided was undeclared for the majority of diets (77%), however, this was assumed to represent the typical analysis in line with FEDIAF guidelines. The guaranteed analysis reports minimum and maximum values, whereas a typical analysis represents exact values.^40^ Given that the majority of manufacturers did not explicitly state the type of analysis utilized, it is challenging to definitely confirm that caloric distribution calculations were based on exact values, as opposed to minimum and maximum values. Whilst this could have contributed to inaccuracies in the calculated metabolizable energy density, both analytical methods were considered acceptable for the purpose of this study.

One of the main limitations of our study was that only the main diet of each dog was analyzed. This was because of the challenges associated with owner recollection of the diet history at the onset of initial clinical signs, which ranged from 1 month to 3 years and 3 days to 3 years in the control dogs and CE dogs, respectively. Whilst treat provisions were recorded as part of this study, it was not possible to confirm their contribution to daily caloric intake for each individual dog and whether this exceeded 10% of daily calories. In people, unbalanced diets are associated with the development of disease and malnutrition, the latter having a subsequent effect on the function of the immune system and microbiota.^41^, ^42^ Similarly, while none of the main diets included carrageenan, CMC, or P80, their presence within additional diets/treats was not assessed. It is therefore challenging to exclude the potential role of these emulsifiers based on the dietary information available from our study. Challenges associated with recall of diet histories by owners, specifically in relation to dogs with gastrointestinal disease, are reported.^43^ Only half of owners can name the specific diet being fed at the time of consultation via a Gastroenterology Service. Obtaining a complete diet history is crucial in identifying potential triggers in the etiopathogenesis of CE in dogs and developing an ongoing nutritional treatment plan. Our study highlights the need for the acquisition of a detailed diet history at the time of consultation to aid future studies examining pre‐illness dietary risk factors. This could then guide the use of preventative measures in predisposed dogs, for example dogs with acute hemorrhagic diarrhea syndrome, which may go on to develop CE.^44^

Our study was also limited by recall bias. The diet history was obtained from the time of onset of initial presenting signs to ensure that the diet analyzed was the one being fed at the start of the disease process. For most owners this required the recollection of a diet fed months to years before being assessed, with many dietary changes occurring over this period. The inclusion of dietary information preceding the start of initial signs would also be helpful in the investigation of pre‐illness dietary risk factors, especially for those cases where the main diet analyzed was fed for a short duration of time. This would have been challenging to execute retrospectively, therefore was not pursued as part of this study.

Both presumptive and confirmed CE cases were included in our study. This reflects a desire to recruit a larger study cohort with a spectrum of disease severity, with those dogs undergoing gastrointestinal biopsies typically having failed dietary management. The dogs in our presumptive CE group did not undergo histopathologic assessment of their gastrointestinal tract or follow‐up to confirm that their enteropathy was dietary responsive. This could have resulted in the misclassification of these cases, however, given the stringent inclusion criteria, excluding all other appreciable underlying causes, this would be considered unlikely.

## CONFLICT OF INTEREST DECLARATION

Authors declare no conflict of interest.

## OFF‐LABEL ANTIMICROBIAL DECLARATION

Authors declare no off‐label use of antimicrobials.

## INSTITUTIONAL ANIMAL CARE AND USE COMMITTEE (IACUC) OR OTHER APPROVAL DECLARATION

Approved by the Clinical Research Ethical Review Board at the Royal Veterinary College, URN SR2020‐0202.

## HUMAN ETHICS APPROVAL DECLARATION

Authors declare human ethics approval was not needed for this study.

## Supporting information


**Data S1.** Supporting Information.
