# Supplementary material for: Pre‐illness dietary risk factors in dogs with chronic enteropathy
Source: J Vet Intern Med. 2023 Sep 24;37(6):2093–101. doi: 10.1111/jvim.16872 (PMC10658591; doi:10.1111/jvim.16872)
Supplement: Supplementary file 1 — Data S1. Supporting Information. [file JVIM-37-2093-s001.pdf]

Supplementary information S1: Copy of the owner questionnaire utilized for data collection.

Global Nutrition Study

The aim of this study is to help advance our knowledge of diet in health and disease in dogs. Therefore, I would be grateful if you are able to answer the following questions regarding diets that have been fed to your pet. By completing the questionnaire, you consent to participate in this study. You do not have to complete it and you can choose not to answer certain questions if you wish, in which case, please leave the question(s) blank.

This questionnaire is anonymous, and your answers will remain confidential. You have the right to withdraw at any time and any data that we have collected will be deleted. Data collected will be stored securely as a hard copy and electronically. All participants will have the right to see a copy of the final report of the study.

- Date questionnaire completed:
- Your pets full name (including surname):
- Please provide the foods that your pet was consuming at the onset of the signs that they are presenting with today to the hospital. *For example, if your pet is presenting today for vomiting, the foods your pet was eating when the vomiting first started.*

Please be as specific as possible when listing the food. For commercial diets, please list the manufacturer, brand, flavor and formulation of the diet. For home-prepared foods, please list the exact cut of meat and how this was prepared (*for example, boneless, skinless chicken thigh, roasted in the oven*). If at all possible, please list the amounts per day that were fed of these foods.

**Commercial diets and commercial treats (please include each on a separate line)**				
Full name of the diet (manufacturer/ brand/formula e.g. Royal Canin Satiety)	Flavor (e.g. chicken)	Formulation (dry/tinned)	Amount fed per day	Approx. how long diet fed prior to onset of signs
1.				
2.				
3.				

**Home-prepared ingredients (please include each on a separate line)**			
Cut of meat (e.g. chicken thigh, skinless, boneless)	Preparation (e.g. roasted)	Amount fed per day	Approx. how long diet fed prior to onset of signs
1.			
2.			
3.			

Supplementary information S2: Questions asked to pet food manufacturers to ascertain adherence to the World Small Animal Veterinary Association Global Nutrition Committee guidelines.

Pet food manufacturers were asked the following 2 initial questions when attempting to ascertain whether they met the World Small Animal Veterinary Association (WSAVA) Global Nutrition Committee (GNC) guidelines. If they were able to answer these 2 questions satisfactorily, the remaining 6 questions outlined in section A of the following web link: **http://www.wsava.org/sites/default/files/Recommendations_on_Selecting_Pet_Foods.pdf** were subsequently asked. Diets were considered to meet the WSAVA GNC guidelines if the manufacturer could satisfactorily answer all 8 questions.

1. *Who formulates the diet?*
   Unless the individuals who formulate the diet had either a PhD or MS in Animal Nutrition, or were Board Certified by the American College of Veterinary Internal Medicine (Nutrition) or the European College of Veterinary Comparative Nutrition (ECVCN), they were considered to not meet the WSAVA GNC guidelines.

2. *What is the quality control process for ingredients and finished products?*
   For example, companies were asked to provide the sodium content on a 100 kcal basis (for the diet in question). If manufacturers could not provide the sodium content on a kcal basis, they were considered to not meet the WSAVA GNC guidelines.

Supplementary information S3: Exclusion characteristics of 7 dogs

A total of 7 dogs were excluded from the caloric distribution analysis due to multiple flavors of the same diet being fed (3/7 dogs), home cooked diets precluding further analysis (3/7 dogs) and a lack of proximate analysis from the manufacturer (1/7 dogs). This also meant that analysis of percentage crude fiber as fed (4/7), percentage moisture as fed (5/7) and percentage crude ash as fed (4/7) was not carried out in these 7 dogs, however, ingredient analysis was still performed. For two commercial raw food diets, the caloric distribution exceeded 100%, one of which was purely meat based, whilst the other contained vegetables. The carbohydrate calculated by difference was negligible (<1.0%) in the latter diet and therefore the output carbohydrate percentage was set to zero for both diets and the percentage protein and percentage fat used for the caloric distribution analysis. However, for the diet with carbohydrate calculated by difference of <1.0%, the diet was still classified as containing carbohydrate for the ingredient analysis.

Supplementary information S4: Demographics of dogs

Five dogs were excluded due to multiple diets being fed and a main diet not being identified. Of the 95 dogs remaining, 47 were control dogs and 48 were CE dogs (25 presumptive and 23 confirmed). The age of the control group ranged from 7 months to 15 years 7 months (median 7 years 7 months) and median body weight was 10.2 kg (range 1.6-41.7). Body condition score was recorded for all dogs (BCS scale 1-9) and categorised as underconditioned (BCS 1-3), adequately conditioned (BCS 4-5) and over conditioned (6-9). Of the 47 control dogs, 2 were underconditioned (4%), 30 were adequately conditioned (64%) and 15 were over conditioned (32%). There were 17 neutered males, 10 intact males, 14 neutered females and 6 intact females. Breeds included 10 cross breed dogs; 3 Staffordshire bull terriers, 3 miniature schnauzers, 3 cocker spaniels; 2 pugs, 2 Pomeranians, 2 German shepherd dogs, 2 dachshunds, 2 chihuahuas, 2 border collies; and 16 individual breeds. The duration of clinical signs ranged from 1 month to 3 years with a median of 6 months in the control dog group. The diagnoses reached included pulmonary disease (15/47 dogs), urogenital disease (7/47 dogs), endocrinopathies (6/47 dogs), neoplastic disease (4/47 dogs), skin disease (3/47 dogs), renal disease (3/47 dogs), dental disease (2/47 dogs), immune mediated disease (2/47 dogs), musculoskeletal disease (2/47 dogs), portosystemic shunt (2/47 dogs) and unknown (1/47 dogs).

Ages of the CE dogs ranged from 3 months to 10 years of age (median 3 years 6 months) and median body weight was 9.6 kg (range 1.9-52). Of the 48 CE dogs, 18 were underconditioned (37%), 22 were adequately conditioned (46%) and 8 were over conditioned (17%). There were 16 neutered males, 14 intact males, 16 neutered females and 2 intact females. Breeds included 12 cross breed dogs, 4 pugs, 4 Labradors, 2 Yorkshire terriers, 2 Staffordshire bull terriers, 2 border terriers and 22 individual breeds. The duration of gastrointestinal signs ranged from 3 days to 3 years with a median of 3 months. Sixteen dogs had a protein-losing enteropathy: 13 in the confirmed CE group and 3 in the presumptive CE group. Two of the 3 dogs with protein-losing enteropathy in the presumptive CE group went into clinical

remission following a therapeutic hydrolyzed protein diet with the one remaining dog achieving partial response to dietary therapy. Histopathologic diagnosis following gastrointestinal biopsy in the confirmed CE dogs included lymphoplasmacytic enteritis (9/23, 39%), lymphoplasmacytic and neutrophilic enteritis (3/23, 13%), plasmacytic enteritis (3/23, 13%), granulomatous colitis (3/23, 13%), lymphoplasmacytic and eosinophilic enteritis (2/23, 9%) and lymphoplasmacytic, eosinophilic, and neutrophilic enteritis (2/23, 9%). All gastrointestinal biopsies were obtained via endoscopy. In one dog no significant abnormalities were noted on gastrointestinal histopathology however, this dog went into clinical remission with a therapeutic hydrolyzed protein diet.

Supplementary information S5: Protein source of diets

Of those diets with a meat-based protein source, poultry was the most frequently encountered (63/92 diets; 36 CE dogs and 27 controls), followed by red meat (17/92 diets; 6 CE dogs and 11 controls), fish (6/92 diets; 3 CE dogs and 3 controls) and mixed meats (6/92 diets; 4 CE dogs and 2 controls). Dogs classified as 'mixed meats' were those that received multiple flavours of the same diet, all of which contained a main protein source of both poultry and red meat. Of the six fish-based diets, only one contained an additional meat source (lamb). Of the diets with a main red meat protein source, 14 (61%) also included poultry or fish, and the remainder were red meat exclusive (9/23, 39%). For the dogs that were fed red meat exclusively, 4/9 contained only lamb, 3/9 contained just beef and 2/9 contained a combination of red meats, consisting of beef, lamb and pork. For those diets classified as non-red meat based on the first listed protein source, a red meat source was noted lower down the ingredients list in 28/72 diets (10 control dogs and 18 CE dogs).

Supplementary information S6: Data for non-significant variables

For both the primary and secondary analysis (Table 1), diet formulation (primary analysis P = .13, secondary analysis P = .06) and dietary processing (primary analysis P = .78, secondary analysis P = .17) were not significant, nor was the presence of wheat in the main diet (primary analysis P = .55, secondary

analysis P = .32). The main diet adhering to the WSAVA GNC guidelines was not significant between the two groups (primary analysis P = .25, secondary analysis P = .08). This was also the case with a source of omega 3 within the main diet (primary analysis P = .10, secondary analysis P = .11) and presence of pre/probiotics (primary analysis P = .94, secondary analysis P = .50).
